# Food groups and colorectal cancer risk

**DOI:** 10.1038/sj.bjc.6690206

**Published:** 1999-03

**Authors:** F Levi, C Pasche, C La Vecchia, F Lucchini, S Franceschi

**Affiliations:** Registre Vaudois des Tumeurs, Institut universitaire de médecine sociale et préventive, Falaises 1, Lausanne, 1011, Switzerland; Istituto di Ricerche Farmacologiche ‘Mario Negri’, Via Eritrea 62, 20157 Milano, Italy; Servizio di Epidemiologia, Centro di Riferimento Oncologico (CRO), Via Pedemontana Occ., Aviano (PN), 33081, Italy

**Keywords:** colorectal carcinoma, diet, case–control study, Switzerland

## Abstract

Most studies of diet and colorectal cancer have considered nutrients and micronutrients, but the role of foods or food groups remains open to debate. To elucidate the issue, we examined data from a case–control study conducted between 1992 and 1997 in the Swiss canton of Vaud. Cases were 223 patients (142 men, 81 women) with incident, histologically confirmed colon (*n* = 119) or rectal (*n* = 104) cancer (median age 63 years), linked with the Cancer Registry of the Swiss Canton of Vaud, and controls were 491 subjects (211 men, 280 women, median age 58 years) admitted to the same university hospital for a wide spectrum of acute non-neoplastic conditions unrelated to long-term modifications of diet. Odds ratios (OR) were obtained after allowance for age, sex, education, smoking, alcohol, body mass index, physical activity and total energy intake. Significant associations were observed for refined grain (OR = 1.32 for an increase of one serving per day), and red meat (OR = 1.54), pork and processed meat (OR = 1.27), alcohol (OR = 1.28), and significant protections for whole grain (OR = 0.85), raw (OR = 0.85) and cooked vegetables (OR = 0.69), citrus (OR = 0.86) and other fruits (OR = 0.85), and for coffee (OR = 0.73). Garlic was also protective (OR = 0.32 for the highest tertile of intake). These findings in a central European population support the hypothesis that a diet rich in refined grains and red meat increases the risk of colorectal cancer; they, therefore, support the recommendation to substitute whole grains for refined grain, to limit meat intake, and to increase fruit and vegetable consumption. © 1999 Cancer Research Campaign


*British Journal of Cancer* (1999) **79**(7/8), 1283–1287
© 1999 Cancer Research Campaign
Article no. bjoc.1998.0206

### Food groups and colorectal cancer risk

**F Levi[1], C Pasche[1], C La Vecchia[2], F Lucchini[1] and S Franceschi[3]**

[1]Registre Vaudois des Tumeurs, Institut universitaire de médecine sociale et préventive, Centre Hospitalier Universitaire Vaudois, Falaises 1, 1011 Lausanne, Switzerland; [2]Istituto di Ricerche Farmacologiche 'Mario Negri', Via Eritrea 62, 20157 Milano, and Istituto di Statistica Medica e Biometria, Università degli Studi di Milano, Via Venezian 1, 20133 Milano, Italy; [3]Servizio di Epidemiologia, Centro di Riferimento Oncologico (CRO), Via Pedemontana Occ., 33081 Aviano (PN), Italy

**Summary** Most studies of diet and colorectal cancer have considered nutrients and micronutrients, but the role of foods or food groups remains open to debate. To elucidate the issue, we examined data from a case–control study conducted between 1992 and 1997 in the Swiss canton of Vaud. Cases were 223 patients (142 men, 81 women) with incident, histologically confirmed colon ($n = 119$) or rectal ($n = 104$) cancer (median age 63 years), linked with the Cancer Registry of the Swiss Canton of Vaud, and controls were 491 subjects (211 men, 280 women, median age 58 years) admitted to the same university hospital for a wide spectrum of acute non-neoplastic conditions unrelated to long-term modifications of diet. Odds ratios (OR) were obtained after allowance for age, sex, education, smoking, alcohol, body mass index, physical activity and total energy intake. Significant associations were observed for refined grain (OR = 1.32 for an increase of one serving per day), and red meat (OR = 1.54), pork and processed meat (OR = 1.27), alcohol (OR = 1.28), and significant protections for whole grain (OR = 0.85), raw (OR = 0.85) and cooked vegetables (OR = 0.69), citrus (OR = 0.86) and other fruits (OR = 0.85), and for coffee (OR = 0.73). Garlic was also protective (OR = 0.32 for the highest tertile of intake). These findings in a central European population support the hypothesis that a diet rich in refined grains and red meat increases the risk of colorectal cancer; they, therefore, support the recommendation to substitute whole grains for refined grain, to limit meat intake, and to increase fruit and vegetable consumption.

**Keywords:** colorectal carcinoma; diet, case–control study; Switzerland

Most studies of diet and colorectal cancer have considered the role of nutrients and micronutrients, such as fat, fibres, folate and calcium, ascorbate, carotenoids and other selected antioxidant vitamins or phytoestrogens. However, the role of at least two foods – red meat as a risk factor and vegetables as protective ones – cannot be fully explained in terms of identified macro- or micronutrients (Willett, 1989; Willett et al, 1990; Potter, 1996). Food variety, moreover, has been inversely related to colorectal cancer risk in a few, though not all, studies (Fernandez et al, 1996).

It is relevant, therefore, to consider dietary factors in colorectal carcinogenesis in terms of individual food items or food groups from a European population whose dietary habits are largely at variance with those of North America, where most studies have been conducted. Information on foods may also be more useful in relation to preventive recommendations, because it avoids the difficulties and uncertainties of conversions into nutrients (Manousos et al, 1983; La Vecchia et al, 1988; Franceschi et al, 1997).

We have, therefore, examined the relations between food groups and colorectal cancer using data from a case–control study conducted in the Swiss canton of Vaud, an area with intermediate colorectal cancer rates on a European scale (Levi et al, 1998).

*Received 30 April 1998*
*Revised 28 July 1998*
*Accepted 30 July 1998*

*Correspondence to:* F Levi, Registre Vaudois des Tumeurs, CHUV-Falaises 1, CH-1011 Lausanne, Switzerland

### MATERIALS AND METHODS

A case–control study of colorectal cancer was conducted between January 1992 and June 1997 in the Swiss canton of Vaud. Cases were subjects with incident (i.e. diagnosed within the year before the interview), histologically confirmed colon or rectal cancers (International Classification of Diseases, 9th Revision, 153.0–154.1; World Health Organization, 1976), who had been admitted to the University Hospital of Lausanne, Switzerland. Cases identified and interviewed were linked with the incidence data from the Vaud Cancer Registry (Levi et al, 1997) to verify the correspondence of their sociodemographic characteristics with the general population. The case series comprised 223 patients (142 men, 81 women) with incident colon ($n = 119$) or rectal ($n = 104$) cancers (median age 63 years).

Controls were subjects residing in the same geographical area, whose admission diagnosis was of acute, non-neoplastic diseases, unrelated to obesity or chronic conditions inducing long-term modification of diet. Controls were selected in comparable strata of age. A total of 491 controls (211 men, 280 women) aged < 75 years (range 27–74 years; median age 58 years) were interviewed. They were also admitted to the University Hospital in Lausanne for a wide spectrum of acute conditions, including traumas (31%, mostly sprains and fractures), non-traumatic orthopaedic diseases (18%, mostly low back pain and disk disorders), surgical conditions (32%, mostly abdominal, such as acute appendicitis, kidney stones or strangulated hernia), and miscellaneous other disorders (19%, including acute medical, eye, nose and throat, and skin diseases).

All interviews were conducted in hospital. Less than 15% of subjects approached for interview refused. The structured questionnaire included information on sociodemographic characteristics and lifestyle habits (e.g. smoking, alcohol consumption and physical exercise), anthropometric factors and a problem-oriented medical history.

A food-frequency questionnaire (FFQ), adapted from a validated one (Franceschi et al, 1993; Decarli et al, 1996), was administered by an interviewer to assess subjects' habitual diet, including total energy. Average weekly frequency of consumption of specific foods or food groups, as well as complex recipes (including the most common ones in the diet) during the 2 years before cancer diagnosis or hospital admission (for controls) was determined. Intakes lower than once per week but at least once per month were coded as 0.5.

The FFQ included 79 foods, food groups or recipes grouped into six sections: (i) bread and cereal dishes; (ii) meat, poultry, fish and foods used as meat substitutes; (iii) vegetables (side dishes); (iv) fruit; (v) sweets and desserts, and soft drinks; (vi) milk and dairy products. Another section dealt with alcoholic beverage consumption. At the end of certain sections, one or two summary questions were included concerning all food items or dishes of a certain type (e.g. any type of meat). Several questions aimed at assessing fat intake pattern were also included.

### Data analysis

Food items and recipes were categorized into 14 groups, i.e. milk and dairy products (ten questions), bread and cereal dishes (eight questions), soups (two questions), eggs (two questions), poultry (two questions), red meat (beef, veal, lamb; six questions), pork and processed meat (four questions), fish (three questions), raw vegetables (four questions), cooked vegetables (eight questions), potatoes (two questions), citrus fruit (two questions), other fruit (eight questions), cakes and desserts (six questions). The total weekly frequency of intake of food items and recipes included in the same group was subdivided into approximate tertiles based on the control distribution.

Odds ratios (OR) and the corresponding 95% confidence intervals (CI) were computed, using unconditional multiple logistic regression models (Breslow and Day, 1980). All regression equations included terms for age in quinquennia, years of education, tobacco (never, ex-smokers, current smokers of < 15 and ≥15 cigarettes per day) and alcohol (non-drinkers, drinkers of < 15 and ≥15 drinks per week, and ex-drinkers), body mass index, physical activity and total energy intake.

Food groups significantly related to colorectal cancer were also simultaneously introduced in a single model including meat and vegetables, to allow for possible reciprocal confounding. Intake frequency of these food groups was also introduced as a continuous variable. In these models, the unit of measurement for each food group was set at seven per week. These models give an estimate of the OR relative to an increase of one average serving per day. Using the distribution of the risk factors in the cases and the ORs from the logistic models, population attributable risks were computed, i.e. the proportion of colorectal cancers that would have been avoided if all subjects were in the most favourable intake level. The method described by Bruzzi et al (1985) implies knowledge of the ORs and of the joint distribution of the risk factors in the population of cases only, and can thus be applied to hospital-based case–control studies.

### RESULTS

Table 1 gives the distribution of colon and rectal cancer cases, and the comparison group, according to sex, age group, education and body mass index. Cases were more frequently men, somewhat more educated and of higher body mass index. Allowance for these factors, therefore, was made in all subsequent analyses.

Table 2 gives the upper limit of intake of control tertile of food or beverage groups, and the corresponding multivariate ORs. No significant or meaningful association was observed with milk, soups, eggs, cakes and desserts, alcohol and tea consumption. Significant direct relations were observed for refined grains (OR = 1.8 for the highest tertile of intake), meats, mainly beef and other red meat (OR = 2.2), and pork and processed meat (OR = 2.9). In contrast, significant inverse relations were observed for whole grains (OR = 0.5), raw vegetables (OR = 0.5), cooked vegetables (OR = 0.4), citrus fruit (OR = 0.5), other fruits (OR = 0.5), garlic (OR = 0.3) and coffee (OR = 0.4).

When food groups significantly related to colorectal cancer in this study were introduced in a single model including meat and vegetable consumption, associations persisted for most of them, but were no more significant for refined grain, poultry and citrus fruit (Table 3).

Table 4 gives the ORs – for colorectal cancer, colon and rectal cancer separately – for an increase of one serving per day for each food significantly related to colorectal cancer risk. These were, for

**Table 1** Distribution of 223 cases of colorectal cancer and of 491 controls according to selected characteristics. Vaud, Switzerland, 1992–97

| | Cases | | Controls |
|---|---|---|---|
| | Colon | Rectum | |
| **Characteristic** | **No. (%)** | **No. (%)** | **No. (%)** |
| Sex | | | |
| Male | 75 (63) | 67 (64) | 211 (43) |
| Female | 44 (37) | 37 (36) | 280 (57) |
| Age group (years) | | | |
| <45 | 6(5) | 7(7) | 77(16) |
| 45–54 | 15(13) | 19(18) | 110(22) |
| 55–64 | 33(28) | 36(35) | 149(30) |
| 65–74 | 65(55) | 42(40) | 155(32) |
| Education (years) | | | |
| <9 | 11(9) | 15(14) | 72(15) |
| 9–12 | 70(59) | 52(50) | 283(58) |
| ≥13 | 38(32) | 37(36) | 136(28) |
| Body mass index (kg $m^{-2}$) | | | |
| <25 | 52(44) | 47(45) | 246(50) |
| 25–29 | 47(39) | 42(40) | 187(38) |
| ≥30 | 20(17) | 15(14) | 58(12) |
| Smoking status | | | |
| Never | 54(45) | 46(44) | 249(51) |
| Ex | 38(32) | 30(29) | 76(15) |
| Current | 27(23) | 28(27) | 166(34) |
| Total energy intake (tertiles) | | | |
| 1st (low) | 32(27) | 23(22) | 163(33) |
| 2nd | 39(33) | 35(34) | 162(33) |
| 3rd (high) | 48(40) | 46(44) | 167(34) |
| Physical activity at work (score)[a] | | | |
| Low | 26(22) | 19(18) | 48(10) |
| Medium | 73(61) | 67(64) | 344(70) |
| High | 20(17) | 17(16) | 97(20) |

[a]The sum does not add up to the total because of some missing values.

 © Cancer Research Campaign 1999

**Table 2** Odds ratios (OR) and 95% confidence intervals (CI)[a] of colorectal cancer among 223 cases and 491 controls according to intake tertile of selected food groups. Vaud, Switzerland, 1992–97

| | Intake tertile | | | | | Intake tertile | | | |
|---|---|---|---|---|---|---|---|---|---|
| **Food group** | **1[b]** | **2** | **3** | **$\chi^2$ (trend)** | **Food group** | **1[b]** | **2** | **3** | **$\chi^2$ (trend)** |
| Milk | | | | 1 | Raw vegetables | | | | 1 |
| Upper limit[c] | 4.0 | 12.0 | | | Upper limit[c] | 5.5 | 9.0 | | |
| OR | 1 | 0.97 | 0.72 | 1.63 | OR | 1 | 1.14 | 0.49 | 7.20** |
| (95% CI) | | (0.64–1.49) | (0.45–1.17) | | (95% CI) | | (0.77–1.69) | (0.30–0.78) | |
| Refined grain | | | | | Cooked vegetables | | | | |
| Upper limit[c] | 15.5 | 24.5 | | | Upper limit[c] | 5.25 | 8.75 | | |
| OR | 1 | 2.01 | 1.79 | 5.59* | OR | 1 | 0.60 | 0.41 | 14.29** |
| (95% CI) | | (1.28–3.15) | (1.12–2.87) | | (95% CI) | | (0.39–0.91) | (0.26–0.66) | |
| Whole grain | | | | | Potatoes | | | | |
| Upper limit[c] | 3.5 | 10.0 | | | Upper limit[c] | 2.0 | 4.0 | | |
| OR | 1 | 1.28 | 0.54 | 2.81 | OR | 1 | 1.46 | 1.41 | 1.99 |
| (95% CI) | | (0.86–1.92) | (0.34–0.85) | | (95% CI) | | (0.96–2.21) | (0.85–2.33) | |
| Soups | | | | | Citrus fruits | | | | |
| Upper limit[c] | 0.5 | 1.0 | | | Upper limit[c] | 1.5 | 3.5 | | |
| OR | 1 | 0.86 | 1.21 | 0.71 | OR | 1 | 0.73 | 0.52 | 7.68** |
| (95% CI) | | (0.52–1.42) | (0.80–1.82) | | (95% CI) | | (0.48–1.12) | (0.33–0.83) | |
| Eggs | | | | | Other fruits | | | | |
| Upper limit[c] | 1.0 | 2.5 | | | Upper limit[c] | 6.75 | 12.5 | | |
| OR | 1 | 0.87 | 1.30 | 1.45 | OR | 1 | 0.56 | 0.53 | 8.19** |
| (95% CI) | | (0.55–1.37) | (0.84–2.02) | | (95% CI) | | (0.37–0.87) | (0.34–0.83) | |
| Poultry | | | | | Cakes and desserts | | | | |
| Upper limit[c] | 0.0 | 1.0 | | | Upper limit[c] | 1.0 | 3.75 | | |
| OR | 1 | 1.09 | 1.71 | 4.57* | OR | 1 | 1.18 | 0.84 | 0.51 |
| (95% CI) | | (0.70–1.69) | (1.03–2.83) | | (95% CI) | | (0.78–1.79) | (0.52–1.34) | |
| Red meat | | | | | Garlic | | | | |
| Upper limit[c] | 2.25 | 3.75 | | | Upper limit[d] | | | | |
| OR | 1 | 1.31 | 2.15 | 10.75** | OR | 1 | 0.51 | 0.32 | 20.31** |
| (95% CI) | | (0.83–2.07) | (1.35–3.42) | | (95% CI) | | (0.35–0.74) | (0.18–0.57) | |
| Pork and processed meat | | | | | Alcohol | | | | |
| Upper limit[c] | 2.0 | 3.5 | | | Upper limit[e] | 0.0 | 14.0 | | |
| OR | 1 | 1.23 | 2.91 | 20.31** | OR | 1 | 0.77 | 1.39 | 2.30 |
| (95% CI) | | (0.76–2.01) | (1.81–4.67) | | (95% CI) | | (0.49–1.20) | (0.86–2.25) | |
| Fish | | | | | Coffee | | | | |
| Upper limit[c] | 1.0 | 1.5 | | | Upper limit[c] | 7.5 | 21.0 | | |
| OR | 1 | 1.37 | 0.90 | 0.05 | OR | 1 | 0.64 | 0.41 | 12.55** |
| (95% CI) | | (0.90–2.08) | (0.59–1.37) | | (95% CI) | | (0.43–0.95) | (0.25–0.68) | |
| Cheese | | | | | Tea | | | | |
| Upper limit[c] | 3.75 | 7.0 | | | Upper limit[c] | <1.0 | 7.0 | | |
| OR | 1 | 1.04 | 1.66 | 5.59* | OR | 1 | 0.96 | 0.64 | 2.32 |
| (95% CI) | | (0.67–1.64) | (1.07–2.59) | | (95% CI) | | (0.53–1.65) | (0.39–1.08) | |

[a]Estimates from multiple logistic regression equations including terms for age, sex, education, smoking, alcohol, body mass index, physical activity and total energy intake. [b]Reference category. [c]Servings per week. [d]Low/medium/high. [e]Drinks per week. *$P$ <0.05; **$P$ < 0.01.

all colorectal cancers combined, 1.32 for refined grains, 1.54 for red meat, 1.27 for pork and processed meat, and 1.28 for alcohol; 0.85 for whole grain and bread, 0.85 for raw and 0.69 for cooked vegetables, 0.86 for citrus and 0.85 for other fruits, and 0.73 for coffee. These results were similar when colon and rectal cancers were considered separately although the associations with whole grain, red meat, pork and processed meat were somewhat stronger for colon, and somewhat stronger with alcohol for rectum, and the protection of coffee was stronger that for colon. Likewise, when right and left colon cancers were analysed separately, the results were similar for all items considered, except coffee for which protection was apparently stronger for right-sided neoplasms (OR = 0.53).

### DISCUSSION

The present study confirms that food intake patterns have a role in the risk of colorectal cancer, even after allowance for total energy, and a number of major non-dietary correlates. In particular, this study shows – and further quantifies in a European population – a direct association between colorectal cancer risk and meats, and specifically red meat, and an inverse one between various types of vegetables and fruit. Coffee drinking was protective, and alcohol was associated with a moderately increased risk.

The present findings are consistent with an inverse association between fruit and vegetable intake and colorectal cancer (Potter

© Cancer Research Campaign 1999 

**Table 3** Odds ratios (OR) and 95% confidence intervals (CI)[a] of colorectal cancer among 223 cases and 491 controls for selected food groups, after reciprocal allowance for meat and vegetable intake. Vaud, Switzerland, 1992–97

| | Intake tertile | | | |
|---|---|---|---|---|
| **Food group** | **1**[b] | **2** | **3** | **$\chi^2$ (trend)** |
| Refined grain | | | | 1 |
| OR | 1 | 1.66 | 1.43 | 1.75 |
| (95% CI) | | (1.04–2.65) | (0.87–2.35) | |
| Poultry | | | | |
| OR | 1 | 1.00 | 1.61 | 3.50 |
| (95% CI) | | (0.64–1.58) | (0.96–2.69) | |
| Red meat | | | | |
| OR | 1 | 1.27 | 2.06 | 9.26** |
| (95% CI) | | (0.81–2.02) | (1.29–3.30) | |
| Pork and processed meat | | | | |
| OR | 1 | 1.12 | 2.33 | 11.76** |
| (95% CI) | | (0.68–1.85) | (1.42–3.83) | |
| Cheese | | | | |
| OR | 1 | 0.90 | 1.57 | 4.48* |
| (95% CI) | | (0.57–1.43) | (0.99–2.47) | |
| Raw vegetables | | | | |
| OR | 1 | 1.21 | 0.55 | 4.08* |
| (95% CI) | | (0.81–1.82) | (0.33–0.90) | |
| Cooked vegetables | | | | |
| OR | 1 | 0.59 | 0.43 | 12.83** |
| (95% CI) | | (0.39–0.90) | (0.27–0.69) | |
| Citrus fruits | | | | |
| OR | 1 | 0.80 | 0.65 | 3.16 |
| (95% CI) | | (0.52–1.23) | (0.40–1.05) | |
| Other fruits | | | | |
| OR | 1 | 0.59 | 0.63 | 8.19** |
| (95% CI) | | (0.38–0.92) | (0.39–1.01) | |
| Garlic | | | | |
| OR | 1 | 0.50 | 0.39 | 14.78** |
| (95% CI) | | (0.34–0.74) | (0.21–0.70) | |
| Coffee | | | | |
| OR | 1 | 0.69 | 0.44 | 9.86** |
| (95% CI) | | (0.44–1.05) | (0.26–0.74) | |

[a]Estimates from multiple logistic regression equations including terms for age, sex, education, smoking, alcohol, body mass index, physical activity, meat and vegetable consumption, and total energy intake. [b]Reference category. *$P$ <0.05; **$P$ < 0.01.

and Steinmetz, 1996; La Vecchia and Tavani, 1998). Although the underlying biological mechanism(s) remain open to discussion in terms of transit time, bile acid binding, or of specific protective agents, such as antioxidants, phenols, indoles, flavonoids, etc. (Potter and Steinmetz, 1996), it is now clear that this protection is shared by a wide spectrum of fruits and vegetables, and is probably due to substances that are thermoresistant (Franceschi et al, 1997).

Of specific interest is the inverse relation with garlic, which has been related to reduced risk of gastric cancer in studies conducted in China (You et al, 1989) and Italy (Buiatti et al, 1989) and of colorectal cancer in China (Hu et al, 1991). This has been linked to selected constituents of garlic, such as allyil sulfides, to an antibacterial or a more specific chemopreventive role of garlic (Dorant et al, 1993). The present data on colorectal cancer, although preliminary, suggest a potentially broader favourable effect of garlic on digestive tract carcinogenesis.

The present study also confirms, and further quantifies, that wholegrain, but not refined grain, intake is inversely related to colorectal cancer risk, thus pointing to a protective role of fibres on colorectal carcinogenesis (Jacobs et al, 1995; Chatenoud et al, 1998).

Red meat has been related to colorectal cancer risk in studies conducted in North America, and the association has been explained in terms of its fat content, bile acid production or by carcinogens developed by cooking (Willett, 1989; Willett et al, 1990). Studies in Europe, where meat consumption is lower than in North America, have shown no consistent association (Goldbohm et al, 1994; Franceschi et al, 1997), and the issue remains open to discussion (Giovannucci et al, 1994). Of particular interest is the strong relation with pork, sausages and processed meats, as found in a prospective study from Norway (Gaard et al, 1996).

Coffee drinking has been inversely related to colorectal cancer in several studies (La Vecchia, 1993; Tavani et al, 1997). The present data also suggest an inverse relation with tea.

A moderate association, suggested by this study, between alcohol drinking and colorectal cancer is in agreement with epidemiological evidence on the issue, with an overall relative risk of 1.1 from a meta-analysis of published data (Longnecker et al, 1990; Doll et al, 1993).

This study is open to some of the criticisms of hospital-based studies (Breslow and Day, 1980). However, the link between its case series and a cancer registration system (Levi et al, 1997), the satisfactory participation of cases and controls, and the inclusion of only acute conditions unrelated to long-term diet modifications in the comparison group are reassuring with respect to possible selection bias. With reference to information bias and confounding, in the same, structured way a validated food frequency questionnaire was administered, and we were able to allow for a measure of total energy intake (Willett and Stampfer, 1986) besides alcohol, tobacco and physical activity and potential sociodemographic correlates of colorectal cancer[1]. Further, most associations persisted after allowance for meat and vegetable intake, i.e. two of the best recognized dietary correlates of colorectal cancer (Willett, 1989).

Because cases were representative of incident cases in this population, we were able to derive population attributable risks (Bruzzi et al, 1985). These were 41% for low intake of whole grain, 35% for high intake of refined grain, 34% for red meat, 38% for low intake of vegetables, 26% for fruits, and 6% for high alcohol intake. The combination of low whole grain, vegetables and fruit with high red meat intake imply an attributable risk of 80%, suggesting a potentially large scope for preventing this common neoplasm in a European population.

### ACKNOWLEDGEMENTS

The contributions of Mrs G Bidois for data collection and checking, of the Vaud Cancer Registry's staff (N Chappuis, S Cotting, N Menoud and VC Te), and of S Gallus for assisting in data analysis, are gratefully acknowledged.

This work was partly supported by the Swiss National Science Foundation (grant no. 32-31330.91) and by the Swiss Cancer Research Fund (KFS, contract AKT no. 72). The authors wish to thank the following hospital physicians for their assistance: H Bossart, P Burckhardt, G Chapuis, P De Grandi, N De Tribolet, J-F Delaloye, D Egloff, E Frenk, M Gillet, S Krupp, F Lejeune,

*© Cancer Research Campaign 1999*

**Table 4** Odds ratios and corresponding 95% confidence intervals (CI)[a] among 223 cases of colorectal cancer and 491 controls for an intake increase of one serving per day of selected food groups. Vaud, Switzerland, 1992–97

| Food group | Odds ratio (95% CI) for Colon cancer | Rectal cancer | Colon and rectal cancers |
|---|---|---|---|
| Refined grain | 1.46(1.20–1.78) | 1.21(0.99–1.49) | 1.32(1.12–1.56) |
| Whole grain | 0.92(0.80–1.07) | 0.86(0.72–1.02) | 0.85(0.75–0.97) |
| Red meat | 1.63(1.30–2.04) | 1.50(1.20–1.88) | 1.54(1.28–1.85) |
| Pork and processed meat | 1.34(1.17–1.53) | 1.18(1.02–1.37) | 1.27(1.13–1.43) |
| Cheese | 1.10(0.99–1.22) | 1.07(0.94–1.21) | 1.09(0.98–1.22) |
| Raw vegetables | 0.90(0.76–1.07) | 0.84(0.69–1.01) | 0.85(0.74–0.98) |
| Cooked vegetables | 0.69(0.54–0.88) | 0.78(0.61–0.99) | 0.69(0.57–0.83) |
| Citrus fruit | 0.90(0.79–1.03) | 0.84(0.72–0.98) | 0.86(0.78–0.96) |
| Other fruits | 0.84(0.71–0.99) | 0.87(0.74–1.03) | 0.85(0.75–0.96) |
| Alcohol | 1.22(1.04–1.43) | 1.38(1.16–1.63) | 1.28(1.11–1.48) |
| Coffee | 0.71(0.55–0.92) | 0.79(0.62–1.00) | 0.73(0.60–0.88) |

[a]Adjusted for age, sex, education, smoking, alcohol, body mass index, physical activity and total energy intake.

P-F Leyvraz, S Leyvraz, J-J Livio, R Mirimanoff, Ph Monnier, P Nicod, R Panizzon (Centre Hospitalier Universitaire Vaudois, Lausanne), and L Zografos (Hôpital Ophtalmique, Lausanne).

### REFERENCES

Breslow NE and Day NE (1980) The analysis of case–control studies. In *Statistical Methods in Cancer Research*, Vol I. IARC Scientific Publication 32: Lyon

Bruzzi P, Green SB, Byar DP, Brinton LA and Schairer C (1985) Estimating the population attributable risk for multiple risk factors using case–control data. *Am J Epidemiol* **122**: 904–914

Buiatti E, Palli D, Decarli A, Amadori D, Avellini C, Bianchi S, Biserni R, Cipriani F, Cocco P, Giacosa A, Marubini E, Puntoni R, Vindigni C, Fraumeni Jr J and Blot W (1989) A case–control study of gastric cancer and diet in Italy. *Int J Cancer* **44**: 611–616

Chatenoud L, Tavani A, La Vecchia C, Jacobs Jr DR, Negri E, Levi F and Franceschi S (1998) Whole grain food intake and cancer risk. *Int J Cancer* **77**: 24–28

Decarli A, Franceschi S, Ferraroni M, Gnagnarella P, Parpinel MT, La Vecchia C, Negri E, Salvini S, Falcini F and Giacosa A (1996) Validation of a food-frequency questionnaire to assess dietary intakes in cancer studies in Italy. Results for specific nutrients. *Ann Epidemiol* **6**: 110–118

Doll R, Forman D, La Vecchia C and Woutersen R (1993) Alcoholic beverages and cancers of the digestive tract and larynx. In *Health Issues Related to Alcoholic Consumption*, 126–166. ILSI Press: Washington

Dorant E, Van den Brandt PA, Goldbohm RA, Hermus RJJ and Sturmans F (1993) Garlic and its significance for the prevention of cancer in humans: a critical review. *Br J Cancer* **67**: 424–429

Fernandez E, D'Avanzo B, Negri E, Franceschi S and La Vecchia C (1996) Diet diversity and the risk of colorectal cancer in Northern Italy. *Cancer Epidemiol Biomarkers Prev* **5**: 433–436

Franceschi S, Negri E, Salvini S, Decarli A, Ferraroni M, Filiberti R, Giacosa A, Talamini R, Nanni O, Panarello G and La Vecchia C (1993) Reproducibility of an Italian food frequency questionnaire for cancer studies: results for specific food items. *Eur J Cancer* **29A**: 1–10

Franceschi S, Favero A, La Vecchia C, Negri E, Conti E, Montella M, Giacosa A, Nanni O and Decarli A (1997) Food groups and risk of colorectal cancer in Italy. *Int J Cancer* **72**: 56–61

Gaard M, Tretli S and Loken EB (1996) Dietary factors and risk of colon cancer: a prospective study of 50 535 young Norwegian men and women. *Eur J Cancer Prev* **5**: 445–454

Giovannucci E, Rimm EB, Stampfer MJ, Colditz GA, Ascherio A and Willett WC (1994) Intake of fat, meat, and fiber in relation to risk of colon cancer in men. *Cancer Res* **54**: 2390–2397

Goldbohm RA, Van Den Brandt PA, Van't Veer P, Brants HAM, Dorant E, Sturmans F and Hermus RJJ (1994) A prospective cohort study on the relation between meat consumption and the risk of colon cancer. *Cancer Res* **54**: 718–723

Hu J, Liu Y, Yu Y, Zhao T, Liu S and Wang Q (1991) Diet and cancer of the colon and rectum: a case–control study in China. *Int J Epidemiol* **20**: 362–367

Jacobs DR, Slavin J and Marquart L (1995) Whole grain intake and cancer: a review of the literature. *Nutr Cancer* **24**: 221–229

La Vecchia C (1993) Coffee and cancer epidemiology. In *Caffeine, Coffee and Health*. Garattini S (ed.), pp. 379–398. Raven Press: New York

La Vecchia C and Tavani A (1998) Fruit and vegetables, and human cancer. *Eur J Cancer Prev* **7**: 3–8

La Vecchia C, Negri E, Decarli A, D'Avanzo B, Gallotti L, Gentile A and Franceschi S (1988) A case–control study of diet and colo-rectal cancer in northern Italy. *Int J Cancer* **41**: 492–498

Levi F, Te VC, Randimbison L and La Vecchia C (1997) Statistics from the Registry of the Canton of Vaud, Switzerland, 1988–1992. In *Cancer Incidence in Five Continents*, vol VII. Parkin DM, Whelan SL, Ferlay J, Raymond L and Young J (eds), pp. 686–689. IARC Scientific Publication 143: Lyon

Levi F, Lucchini F, Boyle P, Negri E and La Vecchia C (1998) Cancer incidence and mortality in Europe, 1988–92. *J Epidemiol Biostat* **3**: 295–361

Longnecker MP, Orza MJ, Adams ME, Vioque J and Chalmers TC (1990) A meta-analysis of alcoholic beverage consumption in relation to risk of colorectal cancer. *Cancer Causes Control* **1**: 59–68

Manousos O, Day NE, Trichopoulos D, Gerovassilis F, Tzonou A and Polychronopoulou A (1983) Diet and colorectal cancer: a case–control study in Greece. *Int J Cancer* **32**: 1–5

Potter J (1996) Nutrition and colon cancer. *Cancer Causes Control* **7**: 127–146

Potter JD and Steinmetz K (1996) Vegetables, fruit and phytoestrogens as preventive agents. In *Principles of Chemoprevention*. Stewart BW, McGregor D and Kleihues P (eds.) pp. 61–90. IARC Scientific Publication 130: Lyon

Tavani A, Pregnolato A, La Vecchia C, Negri E, Talamini R and Franceschi S (1997) Coffee and tea intake and risk of cancers of the colon and rectum: a study of 3,530 cases and 7,057 controls. *Int J Cancer* **73**: 193–197

Willett WC (1989) The search for causes of breast and colon cancer. *Nature* **338**: 389–394

Willett WC and Stampfer MJ (1986) Total energy intake: implications for epidemiologic analyses. *Am J Epidemiol* **124**: 17–27

Willett WC, Stampfer MJ, Colditz G, Rosner BA and Speizer FE (1990) Relation of meat, fat and fiber intake to the risk of colon cancer in a prospective study among women. *N Engl J Med* **323**: 1664–1672

World Health Organization (1976) *International Classification of Diseases for Oncology* (ICD-O), p. 131. World Health Organization: Geneva

You WC, Blot WJ, Chang YS, Ershom A, Yang ZT, An Q, Henderson BE, Fraumeni JF Jr and Wang TC (1989) Allium vegetables and reduced risk of stomach cancer. *J Natl Cancer Inst* **81**: 162–164

*© Cancer Research Campaign 1999*
